# Novel Fluorescence Arginine Analogue as a Sensor for Direct Identification and Imaging of Nitric Oxide Synthase-like Enzymes in Plants

**DOI:** 10.1038/srep32630

**Published:** 2016-09-02

**Authors:** Kang Chang, Tongtong Guo, Pengfei Li, Yin Liu, Yufang Xu, Yuda Fang, Xuhong Qian

**Affiliations:** 1 State Key Laboratory of Bioreactor Engineering, Shanghai Key Laboratory of Chemical Biology, School of Pharmacy, East China University of Science and Technology, Shanghai 200237, China; 2 National Key Laboratory of Plant Molecular Genetics; Shanghai Institute of Plan Physiology and Ecology; Shanghai Institutes for Biological Sciences; Chinese Academy of Sciences; Shanghai, China

## Abstract

Nitric oxide synthase like enzyme (NOS-like enzyme), which produces nitric oxide, participates in many biological processes. However it remains unidentified and highly controversial that plants do possess a NOS-like enzyme. In this paper, a novel arginine analogue NP1 was designed and developed for the direct identification and real time tracking of NOS-like enzymes in plant by fluorescence sensing. It could bind NOS-like enzyme efficiently and enter the cell successfully. *In vivo* fluorescence response results directly proved that NOS-like enzymes did exist in tobacco leaf and would be stimulated by pathogen infection, which also provided a useful chemical tool for the study of the function of NOS-like enzyme in plants.

Nitric oxide (NO) is an important endogenous gaseous signalling molecule which universally exists in animals, plants and microbes. In animals, it regulates a variety of processes ranging from the control of blood pressure and smooth muscle relaxation to immune activation and neuronal signaling^1^. In plants, it modulates the growth and development, such as seed germination, stomatal closure, photosynthesis, respiration, cell death and stress resistance etc.^2^,^3^. When plants are attacked by biotic or abiotic stress, the signal molecule NO is produced, induces the production of other signal molecules including salicylic acid (SA), jasmonic acid (JA), ethylene (ET) and abscisic acid (ABA) etc.^3^,^4^,^5^, which participate in plant immunity including systemic acquired resistance (SAR) and induced systemic resistance (ISR)^6^,^7^,^8^. In animals, nitric oxide (NO) is usually produced by nitric oxide synthases (NOS)^9^, which has three different isoforms including inducible nitric oxide synthase (iNOS), endothelial nitric oxide synthase (eNOS) and neuronal nitric oxide synthase (nNOS)^1^. They catalyze the oxidation of L-arginine leading to L-citrulline and NO^9^. However, the generation of NO in plants is relatively complicated, the origin of NO can be grouped into two pathways: the nitrate reductase (NR) pathway and L-arginine dependent pathway^4^. In L-arginine pathway, plants also require nitric oxide synthase like enzyme (NOS-like enzyme). As one of the most important enzymes producing NO, NOS-like enzyme plays a vital role in plant resistance. It acts at the upstream of signalling pathway, participating in the establishment of SAR and ISR. However, the NOS-like enzyme remains unidentified in plants and the hypothesis that plants do possess a NOS-like enzyme is highly controversial^10^,^11^. But when the mammalian NOS inhibitors were applied in plants, the arginine-dependent NO synthesis activity was inhibited, and the production of NO significantly decreased, the expression of pathogen related-1 (PR-1) protein was also inhibited^12^,^13^. These implied the existence of NOS-like enzyme in plants. With increasing interest in exploring the existence of NOS-like enzyme and its function in plants, sensitive and selective detection techniques for monitoring endogenous NOS-like enzyme are urgently desirable, since the enzyme events in plant growth and development are still not fully understood. The current approaches for NOS-like enzyme detection in plants mainly are immunoassays and immunocytochemical analyses^10^,^14^. However, the antibodies against mammalian NOS may recognize many other proteins not connected with NOS. These methods were also too time-consuming to be applied to prove the existence of NOS-like enzyme in plants. Therefore, it’s of significant importance to find more convenient methods for the direct identification of NOS-like enzyme in plants. Based on these, we choose fluorescence-based assays as a new chemical method for the direct identification and real time tracking of NOS-like enzymes in plant. As we know, fluorescence-based assays could offer convenience, high sensitivity, non-destructiveness, as well as real-time imaging^15^. And fluorescence-based techniques have been widely applied in live cell imaging, medical imaging, drug screening, environmental monitoring and other fields^16^,^17^,^18^. Several small-molecule fluorescent sensors have been applied successfully in zooblast^19^,^20^,^21^,^22^. For example, Eric Deprez *et al*. have reported a two-photon fluorescent sensor which was used for the biological imaging of eNOS in living human umbilical vein endothelial cells^23^. However, there were much fewer reports about fluorescent sensors used in plants, one reason is that the existence of the plant cell wall, small molecule fluorescent sensor is difficult to enter the plant cell.

Herein, we expected to develop a fluorescent sensor which could be successfully used for the direct identification and real time tracking of NOS-like enzyme in plants. The designed fluorescent sensor **NP1** consisted of three moieties: a fluorophore, a quencher and a recognition receptor. In order to recognize NOS-like enzyme in plant efficiently, several NOS inhibitors were selected as recognition receptor^24^,^25^. According to the values of IC_50_ of different inhibitors (Supplementary Table S1), L-NNA is the most efficient NOS inhibitor, but according to the report^26^, the methyl ester of L-NNA, N-Nitro-L-arginine methyl ester (L-NAME) is more potent, possibly because of increased bioavailability^11^. So, L-NAME was chosen as the binding moiety for the recognition of NOS-like enzyme in plants. Fluorophores with absorption and emission in the near-infrared (NIR) region are tend to be selected in living animals due to interference of biomolecules^27^. By contrast, in plants, fluorophores with emission less than 650 nm are more desirable for *in vivo* imaging in order to avoid the interference of the fluorescence of chlorophyll^28^. So, in order to realize the real time tracking of NOS-like enzyme in plants, a 2′,7′-dichlorofluorescein (DCF) fluorophore with emission less than 600 nm was chosen to give the fluorescence signal. Based on these, we illustrated our strategy in Fig. 1. A 2′,7′-dichlorofluorescein (DCF) fluorophore coupled with a piperazine ring as an electron donor (quencher) was linked with the recognition receptor, N-Nitro-L-arginine methyl ester (L-NAME), which is a selective NOS inhibitor and widely used in the research of NOS-like enzyme both in mammals and plants^29^. In a free state, **NP1** kept the prior conformation in which the distal nitrogen atom of piperazine ring was proximal to xanthene ring of the fluorophore, causing the efficient quenching of the fluorescence. Upon the receptor of L-NAME combined with NOS-like enzyme, the conformation of piperazine would change from “boat form” to “chair form”, leading to the nitrogen atom away from the xanthene ring, thus photo-induced electron transfer (PET) effect was quenched and the fluorescence would be recovered. With this fluorescent arginine analogue **NP1** in hand, we can realize the direct identification and tracking of NOS-like enzyme in plants by fluorescence sensing using the mechanism of conformational photo-induced electron transfer (PET)^10^,^14^,^30^.

## Results and Discussion

### Synthesis and characterization of NP1

The synthetic route toward **NP1** is depicted in Supplementary Figure S1. Starting from 2′,7′-dichlorofluorescein, it reacted with allyl bromide to generate D-1, then rearranged to D-2 in diphenyl ether at the condition of reflux, then D-3 was formed by the condensation of D-2, formaldehyde and 1-Boc-piperazine, then deprotected by trifluoroacetic acid to prepare D-4, then by the condensation with p-fluorobenzaldehyde to prepare DCF1, finally, **NP1** was synthesized from DCF1 and L-NAME by condensation and reduction. **NP1** was characterized by ^1^H NMR, ^13^C NMR and HRMS (Supplementary Figures S3–S5). Then the designed fluorescent sensor **NP1** was employed for subsequent studies.

### Fluorescent properties of NP1

**Figure 2:**
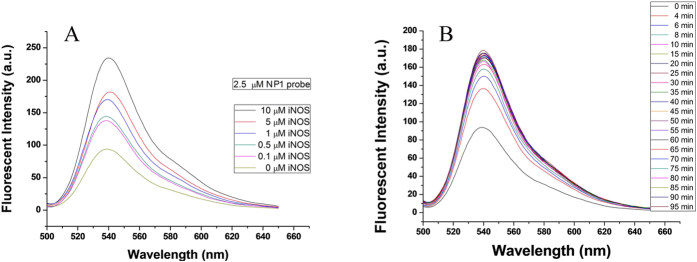
(**A**) The direct fluorescence response of **NP1** to iNOS induced from mouse. Fluorescence responses of **NP1** (2.5 μM) with 0–10 μM iNOS in buffer (50 mM HEPES, pH 7.4, containing <1% DMSO) at 37 °C. Fluorescnce excitation was provided at 480 nm. After adding iNOS to the **NP1** buffer, the fluorescence data were acquired after centain time intervals (100 s) that are indicated in the figure and marked with different colors. (**B**) Time variation in the fluorescence intensity of **NP1** (2.5 μM) at 538 nm with iNOS (5 μM) in buffer (50 mM HEPES, pH 7.4, containing <1% DMSO).

Firstly, in order to determine the excitation and emission wavelength of **NP1**, the spectral characteristics of **NP1** were studied. The pH titration experiments of abosorption and fluorescence were performed in buffer system (50 mM HEPES buffer, pH = 7.4, containing <1% DMSO) at 37 °C. The results showed that the absorption and emission maxima were at 518 nm and 538 nm (Supplementary Figure S2), respectively. In the pH range from 6 to 8, which is consistent with the physiological environment, the emission intensities were quite low and almost unchanged (Supplementary Figure S2). So, **NP1** is quite suitable for imaging in plants. Considering the influence of the shorter Stocks Shift of **NP1** itself, the subsequent fluorescent experiments were performed at an excitation wavelength of 480 nm. Then, the fluorescnece properties of **NP1** (2.5 μM) in the absence and presence of iNOS were determined. Figure 2A depicted elevated fluorescence intensities with increasing the amounts of iNOS (0–10 μM). This was due to the specific binding of **NP1** to iNOS, leading to its conformational change, thereby blocking the PET (Photo electric transfer) quenching. Besides, the dynamic change of fluorescence intensity of **NP1** with iNOS was also tested, as shown in Fig. 2B, the fluorescence intensity in reaction of **NP1** with iNOS reached the maximum value within approximately 10 min. *In vitro* fluorescence data showed **NP1** bound NOS efficiently, and the combination of **NP1** to iNOS was fast and firm, proving that **NP1** was sensitive and stable enough to be applied. With the encouraging data in hands, the imaging experiments in living tobacco leaves were carried out afterwards.

### Detection of NOS-like enzyme in tobacco leaves

**Figure 3:**
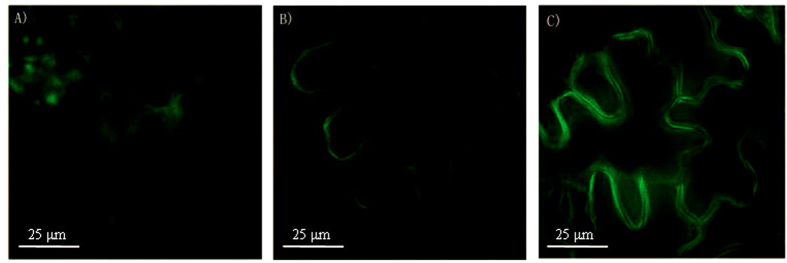
Fluorescence images of tobacco leaf epidermal cells incubated with NP1 (10 μM) containing pathogen of *Pst* DC3000 (OD_600_ = 0.2) or not by syringe injection for 6 h at room temperature taken by a DeltaVision PersonalDV system (Applied Precision) using an Olympus UPLANAPO water-immersion objective lens (60 ×/1.20 numerical aperature)^36^. Filters used for NP1 were exciter (470/40 nm/nm) and emitter (520/40 nm/nm). (**A**) Tobacco leaves incubated with *Pst* DC3000. (**B**) Tobacco leaves incubated with **NP1**. (**C**) Tobacco leaves incubated with **NP1** and *Pst* DC3000.

We thereafter assessed the potential utility of **NP1** to monitor NOS-like enzyme in living tobacco leaves. The four-week old tobacco leaves were chosen for the imaging experiments because of their high endogenous expression of NOS-like enzyme during plant defense^31^. Tobacco leaves injected with the free **NP1** (10 μM) showed relatively weak fluorescence emission (Fig. 3B) after incubated in phytotron for 6 hours. In contrast, upon injected of **NP1** and *Pst* DC3000 to the tobacco leaves at the same time, due to the overexpression of NOS-like enzyme during pathogen infection, a strong green fluorescence was observed (Fig. 3C) after incubated in phytotron for 6 hours. In order to exclude the interference of *Pst* DC3000, we observed the fluorescence of leaves injected with *Pst* DC3000, and found it also showed a weak fluorescence emission (Fig. 3A). This suggested that the fluorescence ehancement was indeed due to the expression of endogenous NOS-like enzyme during pathogen infection.

**Figure 4:**
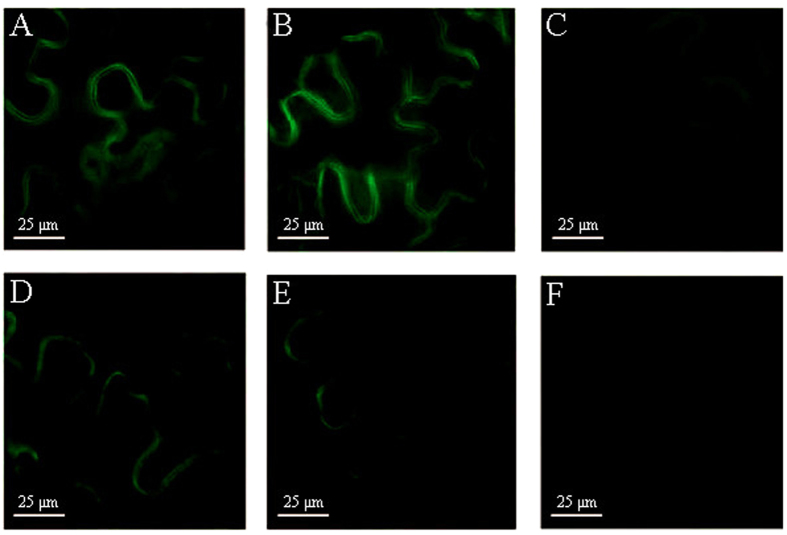
Fluorescence images of tobacco leaf cells incubated with NP1 s(10 μM) containing pathogen of *Pst* DC3000 (OD_600_ = 0.2) or not by syringe injection at room temperature taken by a DeltaVision PersonalDV system (Applied Precision) using an Olympus UPLANAPO water-immersion objective lens (60 ×/1.20 numerical aperature). Filters used for GFP were exciter (470/40 nm/nm) and emitter (520/40 nm/nm). (**A**–**C**) showed tobacco leaves incubated with **NP1** and *Pst* DC3000 for 4 h, 6 h and 8 h, respectively. (**D**–**F**) showed tobacco leaves incubated with **NP1** for 4 h, 6 h and 8 h, respectively.

Subsequently, the dynamic change of fluorescence intensity in tobacco leaves was investigated (Fig. 4) for the study of the burst of NOS-like enzyme during pathogen infection. Fluorescence images were taken in 4 to 8 hours respectively after injection of **NP1** and *Pst* DC3000. A notable fluorescence change was observed, the fluorescence intensity has become stronger 4 hours (Fig. 4A) after the infection of *Pst* DC3000, an increase from 4 to 6 hours (Fig. 4A,B) and a decrease from 6 to 8 hours (Fig. 4B,C) in fluorescence intensity of infected leaves were observed respectively. The decrease of fluorescence intensity may be due to the diffusion of the molecule itself. According to the data, the fluorescence response began 4 hours after infection, much earlier than the onset of SAR which usually expressed PR proteins 1–3 days after invasion^7^. So, the expression of NOS-like enzyme was at the upstream of plant defense and regulated the signal network by producing NO. Therefore, **NP1** could be used as a useful chemical tool for the detection of NOS-like enzyme *in vitro* and in living tobacco leaves, and furthermore, it could also be an indicator to judge the onset of defense response of plants.

**Figure 5:**
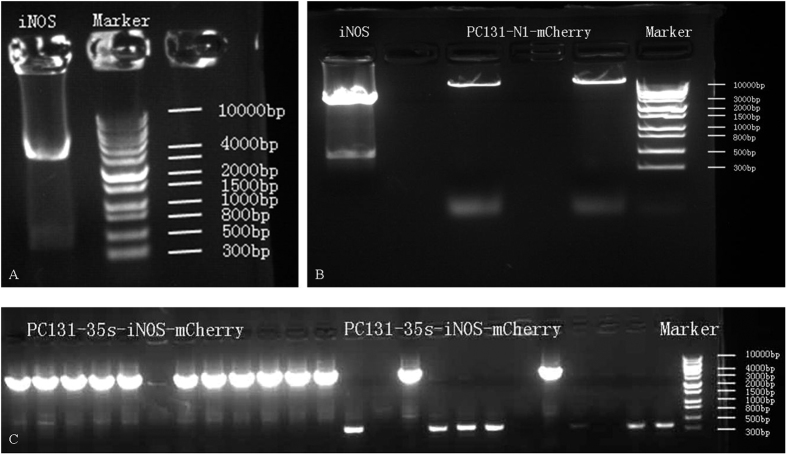
(**A**) The product of iNOS polymerase chain reaction (PCR). (**B**) The product of digestion of iNOS and PC131-35s-N1-mCherry by Sal1 and Spe1. (**C**) The product of colony PCR of PC131-35s-iNOS-mCherry.

**Figure 6:**
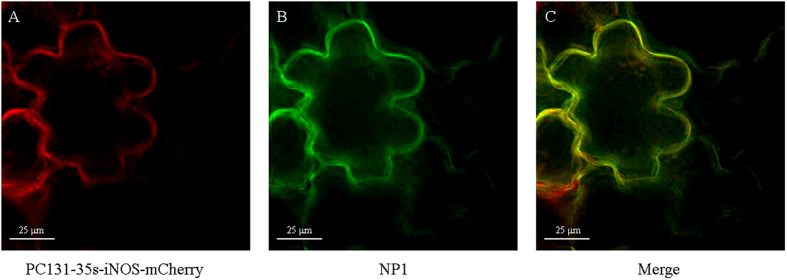
Fluorescence images of tobacco leaf cells incubated with NP1 (10 μM) and PC131-35s-iNOS-mCherry by syringe injection at room temperature taken by a DeltaVision PersonalDV system (Applied Precision) using an Olympus UPLANAPO water-immersion objective lens (60 ×/1.20 numerical aperature). Filters used for images were mCherry and GFP respectively.

Next, in order to further study the subcellular localization of **NP1** and NOS-like enzyme, and the specificity of **NP1** to NOS-like enzyme, a plasmid PC131-35s-iNOS-mCherry including genes of iNOS and a red fluorescent protein named mCherry was constructed^32^,^33^. The length of gene of iNOS was 3438 bp, and the experimental results were consistent with it (Fig. 5). Then it was transformed into agrobacterium tumefaciens and injected into leaves of tobacco. Two days later, the plasmid was expressed completely in tobacco leaves. After that, **NP1** was injected into tobacco leaves, and half an hour later, the images were taken by a fluorescence microscope (Fig. 6). The plasmid was visualized as red fluorescence from the mCherry channel, showing that the inherent localization of NOS-like enzyme in plants was in the cytoplasm of cells (Fig. 6A), and **NP1** was visualized as strong green fluorescence from GFP channel (Fig. 6B). The localization of **NP1** was confirmed by the merged images of the plasmid and **NP1**. It showed a noticeable yellow area (Fig. 6C), indicating that **NP1** was localized in the cytoplasm, where it bonded to iNOS and showed a stronger fluorescence emission.

In summary, we have developed a novel fluorescent arginine analogue **NP1** as a chemical tool for the direct identification and real time tracking of NOS-like enzyme in living tobacco leaves by fluorescence sensing. To our knowledge, this is the first time to use fluorescense sensing for the detection of NOS-like enzyme in plants. Unlike traditional methods, **NP1** was more convenient to be applied and time saving. It enabled fluorescence imaging of endogenous NOS-like enzyme induced by pathogen infection in tobacco leaves, and further proved the existence of NOS-like enzyme in plants. We also found that **NP1** was localized in the cytoplasm and the burst of NOS-like enzyme was at the upstream of defense response in tobacco. Thus **NP1** shows the potential to be used as a valuable research tool in studying biological roles of NOS-like enzyme in plants. Furthermore, **NP1** could also be an indicator to judge the onset of defense response of plants, and in the future, **NP1** has the potential to be exploited as a platform for the screening of plant activators^34^,^35^.

## Methods

### Fluorescent imaging in tobacco leaves

Four-week-old tobacco was chosen for the following experiments. Test condition: **NP1**: 100 μM/L, *Pst* DC 3000: OD_600_ = 0.2. Then **NP1**, *Pst* DC300 and the mix of **NP1** and *Pst* DC3000 were injected into three tobacco leaves by injectors to completely infiltrate leaves. Then incubated in phytotron for 4 hours, and taking images every 2 hours by a fluorescence microscope. Fluorescent image of tobacco leaf cells were acquired at room temperature with a DeltaVision PersonalDV system (Applied Precision) consisting of an IX70 invertedmicroscope (Olympus) equipped with an UPLANAPO water immersion objective lens (60 × 1.20 numerical aperature; Olympus) and a Photometrics (Roper Scientific) CoolSnap ES_2_ camera with Applied Precision customizations and drivers. Filters used for GFP were exciter (470/40 nm/nm) and emitter (520/40 nm/nm).

### Expression of PC131-35s-iNOS-mCherry in tobacco leaves

All enzymes used in this experiment were purchased from TaKaRa Biotechnology Co., Ltd.

#### PCR of iNOS

Because that iNOS is induced from mouse, the cDNA was purchased from Guangzhou FunenGen Co., Ltd. PCR Condition: primer1: 1 μL, primer 2: 1 μL, cDNA: 1 μL, dNTP: 4 μL, H_2_O_2_: 32 μL, primerSTAR HS DNA polymerase: 1 μL, 5 × primerSTAR buffer: 10 μL. After reaction, the result was confirmed by agarose chromatography (Fig. 5A) and then the PCR product was recycled by a kit purchased from Shanghai Generay Biotech Co., Ltd. Then, the product was dissolved in 30 μL water for the following experiments.

#### Digestion of iNOS and PC131-35s-N1-mCherry

Condition for iNOS: iNOS PCR product: 30 μL, Sal 1 enzyme: 0.75 μL, Spe 1 enzyme: 0.75 μL, 10 × H Buffer: 4 μL, H_2_O_2_: 4.5 μL. Condition for PC131-35s-N1-mCherry (vector): vector: 4 μL, Sal 1 enzyme: 0.75 μL, Spe 1 enzyme: 0.75 μL, 10 × H Buffer: 2 μL, H_2_O_2_: 12.5 μL. Putting them in incubator of 37 °C for 2 hours. After that, the mix were separated by agarose chromatography (Fig. 5B) and the right bands were cut off and recycled by corresponding kit purchased from Shanghai Generay Biotech Co., Ltd.

#### Connection of iNOS with vector

Condition: recycled iNOS: 6 μL, recycled vector (PC131-35s-N1-mCherry): 2 μL, T4 ligase: 1 μL, T4 Buffer: 1 μL. Mixing thoroughly, then placing it in incubator of 22 °C for 4 hours. After that, the connection product was put into coli, ice-bath 30 minutes, then stimulated at water bath of 42 °C for 90 seconds, after that, adding 800 μL LB culture medium, and putting it in 37 °C swing bed for 1 hour. Then placing the mix on the culture dish, and cultivated at 37 °C overnight. The connection efficacy was shown in Fig. 5C.

#### Plasmid extraction

Put the bacterial colony into 4 mL LB culture medium with kanamycin, cultivated in 37 °C swing bed overnight. Then extract the plasmid according the corresponding kit purchased from Shanghai Generay Biotech Co., Ltd. And at last, the plasmid was confirmed by sequencing.

#### Transformation

0.6 μL plasmid was added into agrobacterium, mixed thoroughly, stimulated by electric shock. Then, the mix was added into 700 μL LB culture medium, cultivated in 30 °C swing bed for 1 hour. Then the mix was placed on the culture dish, and cultivated at 30 °C for 2 days. Then, the bacterial colony was collected, and suspended in 1 mL water and injected into tobacco leaves with an injector to completely infiltrate leaves. Two days later, the protein was completely expressed in tobacco leaves.

## Additional Information

**How to cite this article**: Chang, K. *et al*. Novel Fluorescence Arginine Analogue as a Sensor for Direct Identification and Imaging of Nitric Oxide Synthase-like Enzymes in Plants. *Sci. Rep.* **6**, 32630; doi: 10.1038/srep32630 (2016).

## Supplementary Material

Supplementary Information

## Figures and Tables

**Figure 1:**
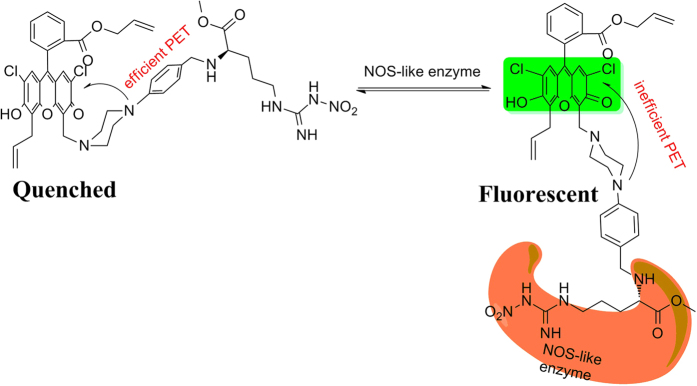
Design idea of NP1 for the recognition of NOS-like enzymes.
