# Supplementary material for: Novel Fluorescence Arginine Analogue as a Sensor for Direct Identification and Imaging of Nitric Oxide Synthase-like Enzymes in Plants
Source: Sci Rep. 2016 Sep 2;6:32630. doi: 10.1038/srep32630 (PMC5009301; doi:10.1038/srep32630)
Supplement: Supplementary Information [file srep32630-s1.pdf]

Supplementary Information

Novel Fluorescence Arginine Analogue as a Sensor for Direct Identification and Imaging of Nitric Oxide Synthase-like Enzymes in Plants

Kang Chang[a],‡ Tongtong Guo[b],‡ Pengfei Li[a], Yin Liu[b], Yufang Xu[a],* Yuda Fang[b],* Xuhong Qian[a]*

1. General experiments

Materials and methods

N, N-dimethylformamide (DMF) was distilled from calcium hydride (CaH_2) under anhydrous conditions. Other chemical regents and solvents were purchased from *J&K* Corporation and used without further perification. All reactions were carried out under a helium atmosphere with analytic grade solvents, unless noted. All reactions were monitored by thin-layer chromatography (TLC) using UV-light (254 nm) and Flu-light (365 nm). Silica gel (300 - 400 mesh) was used for column chromatography.

Instrument

Fluorescence spectra were determined using a Varian Cary Eclipse fluorescence spectrometer. Absorption spectra were determined by a Varian Cary 100 UV-vis spectrophotometer. All pH measurements were made with a Sartorius basic pH-Meter PB-20. ^{1}H NMR and ^{13}C NMR spectra were recorded employing a Bruker AV-400 spectrometer (Me_4Si as internal standard). Mass spectra was performed in a HP 1100 LC-MS spectrometer.

Microscope

Fluorescent image of tobacco leaf cells were acquired at room temperature with a DeltaVision PersonalDV system (Applied Precision) consisting ofanIX70 invertedmicroscope (Olympus) equipped with an UPLANAPO water immersion

objective lens (60×1.20 numerical aperature; Olympus) and a Photometrics (Roper Scientific) CoolSnap ES_2 camera with Applied Precision customizations and drivers[1]. Filters used for GFP were exciter (470/40 nm/nm) and emitter (520/40 nm/nm).

2. Design and synthesis of NP1

Table S1. The inhibitors of NOS-like enzyme

Inhibitor	structure	IC_{50} (μM)		
		iNOS	nNOS	eNOS
L-NNA	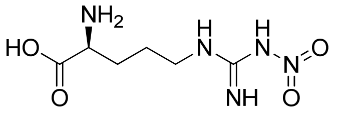	3.1	0.29	0.35
L-NMMA	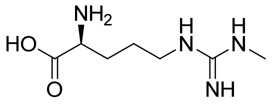	6.6	4.9	3.5
7-NI	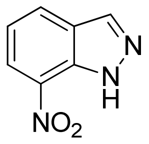	9.7	8.3	11.8
Aminoguanidine	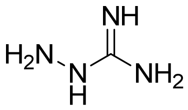	31	170	330
L-NIL	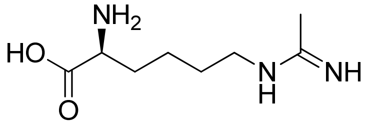	1.6	37	49
1400W	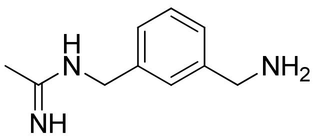	0.23	7.3	1000
GW273629	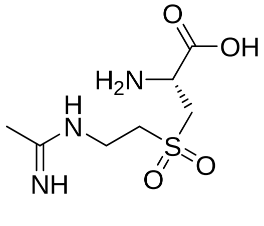	8.0	630	1000

GW274150	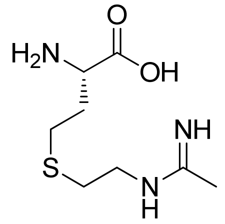	1.4	145	466

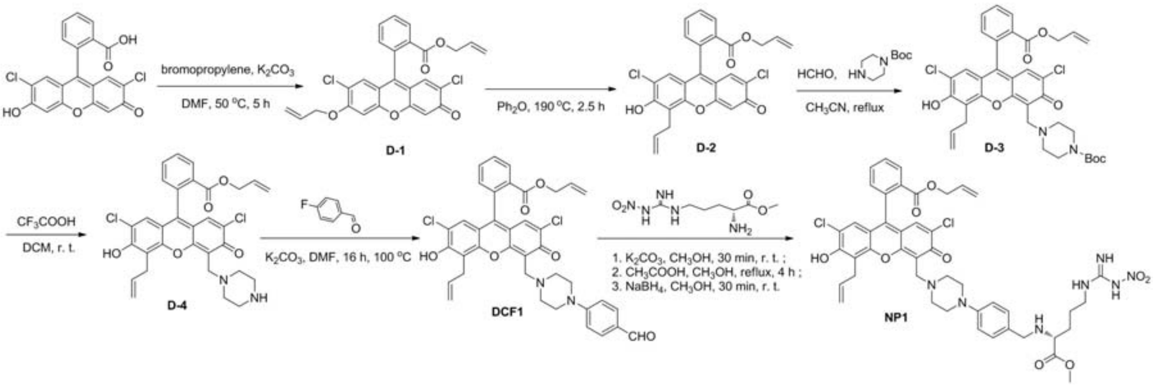

Figure S1. Synthetic route of probe **NP1**

Synthesis of DCF1

Compound **D-4** was prepared according to the literature procedure[2]. A solution of **D-4** (150 mg, 0.25 mmol), potassium carbonate (72 mg, 0.52 mmol) and 4-fluorobenzaldehyde (39 mg, 0.32 mmol) was heated for 16 h in DMF(10 mL) at 100 ^oC. The reaction mixture was poured to water (60 mL), and extracted with acetylacetic eater (60 mL ×2). Subsequently, the organic layer were dried over anhydrous magnesium sulfate, filtered, and concentrated in vacuo. The crude product was purified by column chromatography (silica gel: 120 mL, eluent: 33 % Petroleum ether in EtOAc) to afford a red solid in 56 % yield. ^1H NMR (400 MHz, DMSO-d_6) δ 9.77 (s, 1H), 8.20 (d, J = 7.2 Hz, 1H), 7.88-7.77 (m, 4H), 7.45 (d, J = 7.2 Hz, 1H), 7.14 (d, J = 8.8 Hz, 2H), 6.77 (s, 1H), 6.75 (s, 1H), 5.96-5.90 (m, 1H), 5.66-5.58 (m, 1H), 5.11-5.03 (m, 3H), 4.95 (d, J = 10.0 Hz, 1H), 4.50 (d, J = 5.2 Hz, 2H), 4.41 (m, 2H), 4.49 (d, J = 5.6 Hz, 2H), 3.71 (s, 4H), 3.54 (s, 2H), 3.34 (s, 4H); HRMS (ESI$^+$) calcd for $C_{38}H_{32}Cl_2N_2O_6$ $[M+H]^+$ 683.1710, found 683.1701.

Synthesis of NP1

A solution of **DCF1** (50 mg, 73 μmol), potassium carbonate (50 mg, 360 μmol) and Nω-Nitro-L-arginine methyl ester (42 mg, 180 μmol) was stired for 30 min in MeOH

(10 mL) at room temperature. Subsequently, the reaction mixture was adjusted to pH 5-6 using glacial acetic acid (dropwise) and was refluxed for 4 h, then cooled to room temperature before addition of $NaBH_4$ (10 mg, 260μmol). Stirring was continued at room temperature for 30 min. The reaction mixture was concentrated to remove MeOH in vacuo. The crude product was purified by column chromatography (silica gel: 120 mL, eluent: 2 % MeOH in DCM) to afford a purple solid in 38 % yield. ^{1}H NMR (400 MHz, 20% $CDCl_3$ in CD_3OD) δ 8.32-8.30 (m, 1H), 7.82-7.80 (m, 2H), 7.35-7.32 (m, 1H), 7.26 (d, J=8.4 Hz, 2H), 6.99-6.92 (m, 4H), 6.05-5.98 (m, 1H), 5.67-5.59 (m, 1H), 5.13-5.00 (m, 4H), 4.56 (s, 2H), 4.50 (d, J=5.6 Hz, 2H), 3.77 (s, 1H), 3.74 (s, 3H), 3.70 (br s, 2H), 3.66 (s, 1H), 3.61 (s, 1H), 3.57 (s, 1H), 3.47 (br s, 8H), 3.36 (s, 2H), 3.20 (br s, 2H), 1.65 (br s, 4H); ^{13}C NMR (100 MHz, DMSO-d_6) δ 175.0, 173.0, 172.0, 171.1, 165.7, 165.3, 159.8, 159.7, 154.8, 153.2, 151.0, 149.5, 136.8, 134.4, 133.4, 132.1, 131.1, 131.0, 130.5, 130.3, 129.5, 128.4, 127.1, 126.1, 125.8, 118.4, 116.0, 114.9, 113.9, 109.6, 107.9, 65.8, 59.8, 55.3, 53.1, 52.7, 52.4, 53.3, 52.0, 51.2, 50.6, 49.0, 47.0, 29.8, 28.0; HRMS (ESI$^+$) calcd for $C_{45}H_{47}Cl_2N_7O_9$ $[M+H]^+$ 900.2885, found 900.2863.

3. Bioassay

Effects of pH values

Fluorescence pH titrations were performed in buffer solution at a probe concentration of 10 μM in 50 mM HEPES (containing < 1% DMSO) at 37 °C. As shown in Fig. S2, the absorption and emission maxima of **NP1**were observed at 518 nm and 538 nm, respectively. **NP1** is stable during pH from 6 to 8, while the emission of **NP1** is minimum.

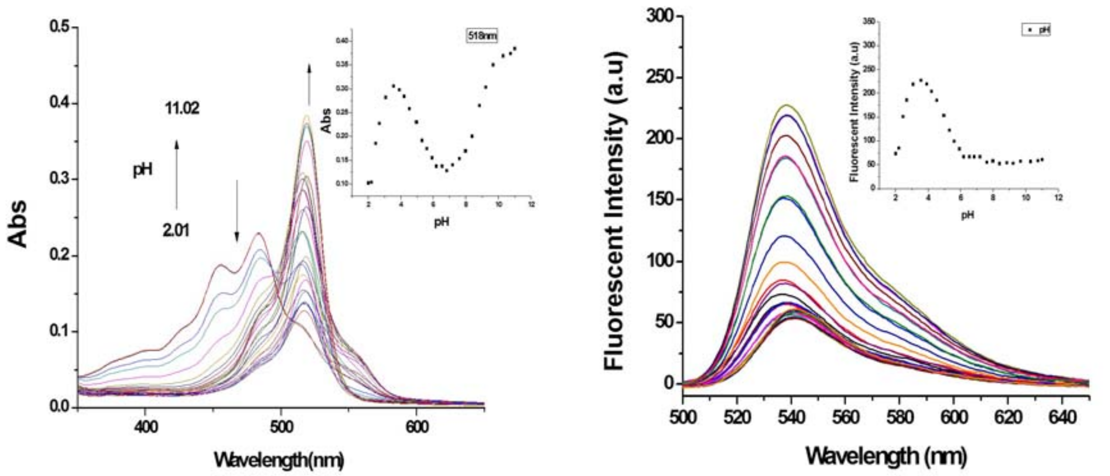

Figure. S2 Absorption and fluorescence response of NP1 (10 μM) to various pH from 2 to 11 in 50 mM HEPES (containing < 1% DMSO) at 37 °C.

4. The characterization data of NP1

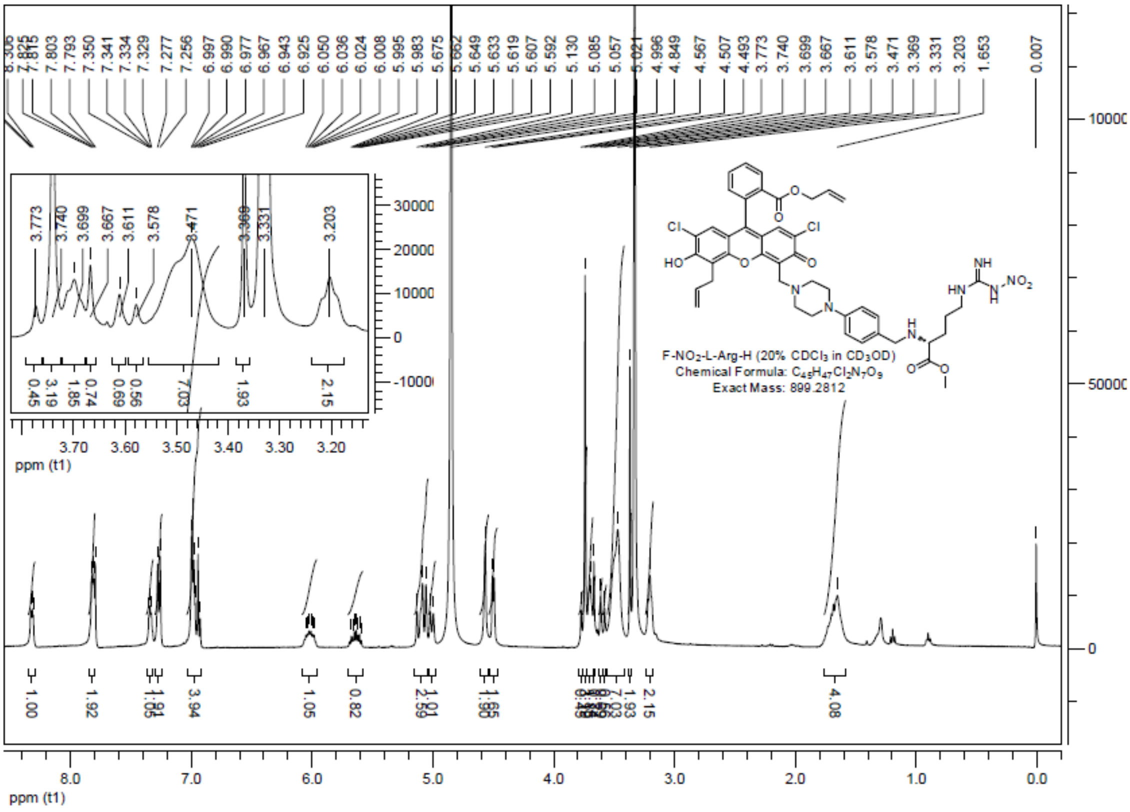

Figure. S3 ^{1}H NMR of **NP1**

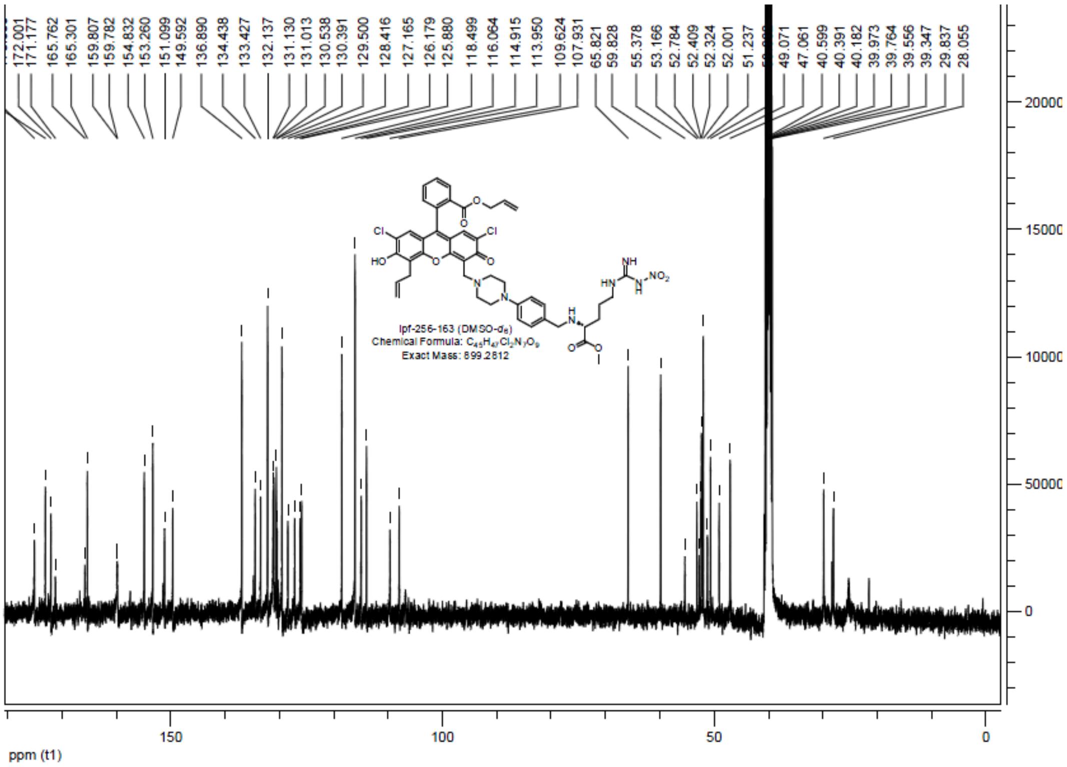

Figure. S4 ^{13}C NMR of **NP1**

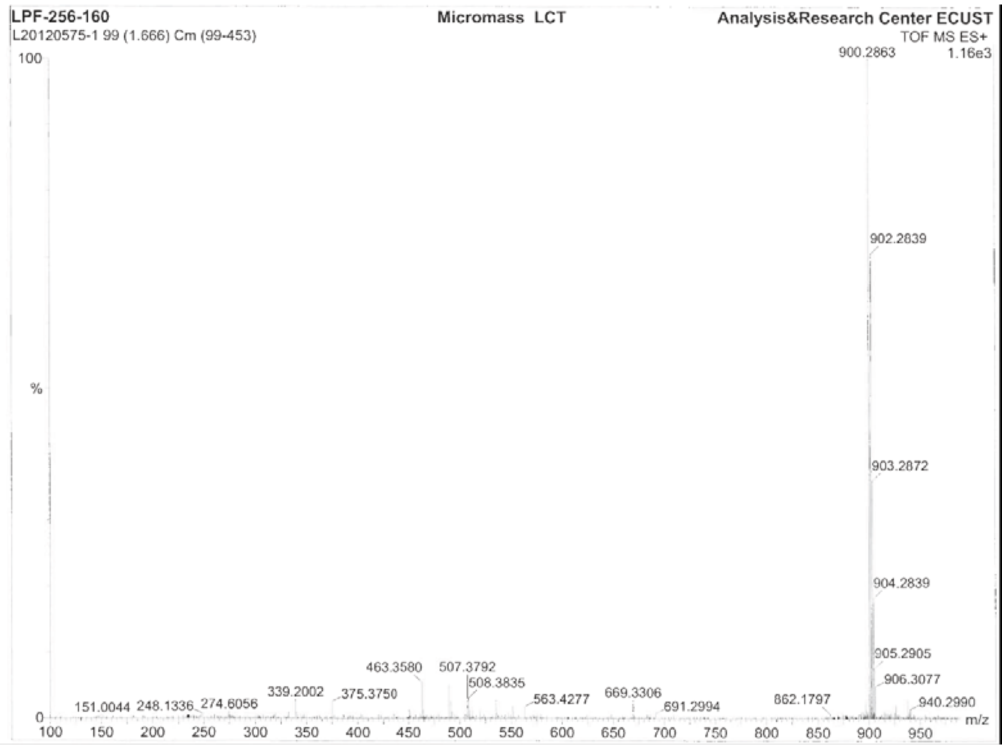

Figure. S5 ESI-Mass spectrum of **NP1**

5. Reference

1 Fang, Y. & Spector, D. L. Live cell imaging of plants. *Cold Spring Harbor protocols* **2010**, pdb top68, doi:10.1101/pdb.top68 (2010).

2 Sparano, B. A., Shahi, S. P. & Koide, K. Effect of Binding and Conformation on Fluorescence Quenching in New 2‘,7‘-Dichlorofluorescein Derivatives. *Org Lett* **6**, 1947-1949, doi:10.1021/ol049537y (2004).
